# The negative association between sodium-driven nutrient pattern and telomere length: the chain mediating role of diastolic pressure and waist circumference

**DOI:** 10.1007/s40520-024-02852-1

**Published:** 2024-10-05

**Authors:** Baodi Xing, Jie Yu, Yiwen Liu, Shuli He, Qi Gao, Xinyue Chen, Fan Ping, Lingling Xu, Wei Li, Huabing Zhang, Yuxiu Li

**Affiliations:** 1 Department of Endocrinology, Key Laboratory of Endocrinology of National Health Commission, Translation Medicine Center, Peking Union Medical College Hospital, Chinese Academy of Medical Sciences and Peking Union Medical College, Beijing, China; 2 Department of Nutrition, Peking Union Medical College Hospital, Chinese Academy of Medical Sciences and Peking Union Medical College, Beijing, China

**Keywords:** Nutrient patterns, Leukocyte telomere length, Principal component analysis, Metabolism, Mediation effect

## Abstract

**Background:**

Numerous single nutrients have been suggested to be linked with leukocyte telomere length (LTL). However, data on nutrient patterns (NPs), particularly in Chinese population, are scarce. This study aimed to examine the relationship between nutrient-based dietary patterns and LTL, and the potential role of metabolic factors.

**Methods:**

Dietary data was obtained via 24-hour food recalls, and principal component analysis (PCA) was used to identify NPs. LTL was assessed using a real-time PCR assay. Multiple linear regression was conducted to determine the association between NPs and LTL. The potential role of metabolism among them was analyzed using mediation models.

**Results:**

A total of 779 individuals from northern China were included in this cross-sectional analysis. Five main nutrient patterns were identified. Adjusted linear regression showed that the “high sodium” pattern was inversely associated with LTL (*B*=-0.481(-0.549, -0.413), *P* < 0.05). The “high vitamin E-fat” pattern exhibited a positive correlation (*B* = 0.099(0.029, 0.170), *P* < 0.05), whereas the “high vitamin A-vitamin B2” pattern was negatively correlated with LTL (*B*=-0.120(-0.183, -0.057), *P* < 0.05), respectively. No significant associations were observed for the remaining nutrient patterns. The mediation model demonstrated that diastolic blood pressure and waist circumference could individually and collectively mediate the negative impact of the “high sodium” pattern on LTL (*B*_DBP_=-0.0173(-0.0333, -0.0041), *B*_WC_=-0.0075(-0.0186, -0.0004), *B*_joint_=-0.0033 (-0.0072, -0.0006), all *P* < 0.05). Moreover, glycosylated hemoglobin and non-high-density lipoprotein cholesterol mediate the relationship between the “high vitamin E-fat” pattern and LTL (*B*_HbA1c_=0.0170(0.0010,0.0347), *B*_non-HDL-C_= 0.0335 (0.0067, 0.0626), all *P* < 0.05), respectively.

**Conclusions:**

The “high sodium” and “high vitamin E-fat” nutrient patterns demonstrated negative and positive associations with LTL and metabolic indicators may play complex mediating roles in these relationships.

**Supplementary Information:**

The online version contains supplementary material available at 10.1007/s40520-024-02852-1.

## Introduction

Telomeres are special DNA-protein complexes at the ends of chromosomes in eukaryotic cells that can protect chromosome ends stability and genome integrity. Telomere attrition occurs each time a somatic cell divides; thus, telomere length (TL) is also considered a primary hallmark of biological aging [1]. Robust evidence has supported that shorter TL is associated with a decrease in life expectancy and the risk of developing several age-related chronic diseases like diabetes mellitus (DM), cardiovascular disease (CVD), and cancer [2, 3]. In recent years, studies have demonstrated that telomere attrition is modifiable, as a substantial variability in telomere shortening rate independent of chronological age [4]. Lifestyle, especially diet, is considered a crucial environmental variable affecting TL [5].

Increasing evidence suggests that nutrient intake is closely related to TL. For instance, higher consumption of dietary vitamin C, vitamin E, β-carotene, and copper may be positively associated with TL, while excessive intake of sodium and iron may be negatively correlated with TL [6–8]. To date, the investigation on the relationship between dietary intake and TL has predominantly focused on single nutrients. However, nutrients are not consumed in isolation, and therefore traditional single-nutrient analysis fails to take into account the complex interactions that occur among them [9]. By contrast, dietary patterns can consider the relationship between the intake and composition of several types of food or nutrients and diseases as a whole, which has more practical significance for guiding nutritional intervention [10]. Previous findings have shown the association between dietary patterns based on food groups and chronic diseases, such as high adherence to the Mediterranean diet, has a protective effect on TL and metabolic diseases; while Western dietary habits, which are characterized by high calories, fat and protein, may have unfavorable impacts on human health [11]. Nevertheless, studies specifically examining the relationship between nutrient-based dietary patterns and aging are limited.

Additionally, as previously reported, metabolic disorders including increased waist circumference (WC), hypertension (HTN), and hyperglycemia have been linked to shorter telomeres. Numerous studies underscore the critical role diet plays in influencing metabolic dysfunction [9, 12]. However, the specific effects of metabolic factors on the relationship between nutrient patterns (NPs) and TL have been less frequently reported.

Thus, this study aimed to analyze the relationship between the nutrient intake model extracted by principal component analysis (PCA) and TL as well as the role of metabolic variables among them in the Chinese population.

## Materials and methods

### Study population

This study is cross-sectional with data from an ongoing type 2 diabetes project in Changping rural communities from Beijing, China, from 2014 to 2020. A total of 1148 participants were invited to complete a questionnaire involving demographic characteristics, diet information, disease and medication history, and a detailed physical examination. Individuals with the following criteria were excluded: (1) with cancer, severe cardiovascular diseases, liver and renal insufficiency; (2) application of drugs interfering with blood glucose, blood pressure, or blood lipids; (3) refusal for LTL test; (4) lack of assessment of HbA1c, and other metabolic variables, as well as complete diet data, and with extreme energy intake (< 500 kcal or > 5000kcal) [13]. Finally, 779 eligible participants were selected for the analysis. (Fig. S1) This study was approved by the Ethics Committee of Peking Union Medical College Hospital and all subjects signed informed consent voluntarily.

### Clinical assessment

Participants were asked to measure weight and height with light clothes and without shoes. Body mass index (BMI) was calculated as body weight divided by height square (kg/m^2^). WC was measured to the nearest 0.1 cm halfway between the iliac crest and the costal margin. After participants rested for 15 min, sitting blood pressure was measured by an experienced physician using a standard mercury sphygmomanometer. The measurement was taken twice consecutively, and the average of the two readings was used for analysis. HTN was defined as systolic blood pressure (SBP) ≥ 140mmHg or diastolic blood pressure (DBP) ≥ 90mmHg, or diagnosed with HTN previously but without hypertension drugs application.

### Biochemical analysis

All participants underwent standardized 75 g-oral glucose tolerance trials (OGTT), which were carried out after overnight fasting (> 10 h), with the blood samples including plasma glucose and insulin collected. According to glucose state, all subjects were classified into normal glucose tolerance (NGT), pre-diabetes mellitus (preDM) (impaired fasting glucose (6.1 ≤ fasting blood glucose (FBG) < 7.0 mmol/L and 2 h blood glucose (2hBG) < 7.8 mmol/L) and/or impaired glucose tolerance (7.8 ≤ 2hBG < 11.1 mmol/L)), and DM [14]. Glycosylated hemoglobin (HbA1c) levels were analyzed by high-performance liquid chromatography (HPLC, intra-assay coefficient of variation (CV) < 3%, inter-assay CV < 10%). Alanine aminotransferase (ALT), aspartate aminotransferase (AST), creatinine, serum uric acid (sUA), and lipid profiles, including triglyceride (TG), total cholesterol (TC), low-density lipoprotein cholesterol (LDL-C) and high-density lipoprotein cholesterol (HDL-C) were evaluated by an automated analyzer (AU5800 automatic biochemical analyzer, Beckman Coulter, USA). Non-HDL-C was defined as TC minus HDL-C. The estimated glomerular filtration rate (eGFR) was accessed using the Chronic Kidney Disease Epidemiology Collaboration equation [15].

### Insulin resistant assessment

The homeostatic model assessment of insulin resistance (HOMA-IR) is a widely used clinical surrogate measure to evaluate insulin resistance (IR): HOMA-IR = FBG (mmol/L) × fasting insulin (FINS) (IU/mL)/22.5. Besides, we also applied two other simple and reliable calculated indexes of IR: triglyceride-glucose (TyG) index and triglyceride to high-density lipoprotein cholesterol ratio (TG/HDL-C) [16]. The formula of TyG index was: Ln ((fasting triglycerides) (mg/dL) ×FBG (mg/dL)/2).

### LTL measurements

Peripheral blood LTL analysis has been described in detail previously [17]. Briefly, the QIAamp DNA blood mid kit (Qiagen, Hilden, Germany) was applied to extract genomic DNA in leukocytes. Purified DNA samples were diluted and quantified using a NanoDrop 1000 spectrophotometer (Thermo Fisher Scientific, Wilmington, DE, USA). LTL was represented as the relative ratio of telomere repeat copy number to the single copy number (T/S) using novel monochrome multiplex quantitative PCR. The within-plate and between-plate CVs were 18% and 7%, respectively. Z scores standardized LTL (z-LTL) was computed and applied for all analyses to minimize the impact of potential batch shift on LTL measurement [18].

### Dietary survey and assessment

Dietary information was collected using the 24-hour food recalls, as documented in our previous studies [19]. The dietary data were reviewed by dietitians and entered into the nutrition calculation software (developed by researchers based on the Microsoft Office Access 2007 database). The food ingredient data was calculated using the China Food Composition Table (2009) database as a guide [20]. Each participant was calculated for the intake of total energy and seventeen nutrients, including carbohydrate, fat, protein, fiber, vitamin A, vitamin B1, vitamin B2, vitamin C, vitamin E, calcium, potassium, magnesium, sodium, iron, selenium, zinc, and copper.

### Dietary patterns

NPs were determined by performing the principal component analysis (PCA). Before extracting the patterns (factors), the correlation matrix of 17 nutrients was examined through Kaiser-Meyer-Olkin (KMO) and Bartlett’s sphericity tests to justify the choice of factors analysis. Both KMO = 0.892 and *P* < 0.001 in Bartlett’s tests indicated that PCA was an appropriate method for the data reduction approach used for the nutrient data in this study. The number of NPs was determined based on the eigenvalues (> 1), the scree plot, and the interpretation of the factors [21]. Varimax rotation was applied using the maximum variance method to obtain nutrient-based factor loadings. Nutrients with loadings greater than ± 0.47 were considered to be major contributors and were used to name the NPs [22]. Within a specific nutrient pattern, higher factor scores indicate a greater adherence to that pattern.

### Statistical analysis

窗体底端.

Z-LTL was categorized into quartiles and the baseline characteristics of participants were depicted and compared across the groups. Continuous variables were presented as means with standard deviation (SD) for normal distribution or medians (interquartile range) for non-normal distribution data. Pairwise comparisons were evaluated using ANOVA for normally distributed data and the Kruskal-Wallis test for non-normally distributed data. Categorical variables were expressed by numerical values (n) and percentages (%) and differences between groups were assessed using the Chi-square test.

Since total energy intake and nutrient pattern scores were non-normally distributed, Spearman correlation analysis was initially used to assess the relationships between the five nutrient patterns, LTL, and total energy intake. Subsequently, partial correlation analysis was conducted to evaluate the associations between nutrient patterns, LTL, and various metabolic variables, while controlling for the potential influence of total energy intake. Furthermore, the association between the five nutrient patterns and LTL was analyzed using generalized linear regression across four models to mitigate the influence of potential confounders. Model 1 was crude model and model 2 was adjusted for total energy intake. Model 3 additionally accounted for the impact of age and sex, beyond total energy intake. In model 4, traditional metabolic factors including WC, SBP, DBP, HbA1c, TG, HDL-C, non-HDL-C, UA, and FINS were further incorporated.

PROCESS macro Version 3.4 [23] was applied to explore whether some metabolic factors mediated the relationship between NPs and LTL. Initially, we used a simple mediation model to identify metabolic variables that might mediate the relationship between NPs and LTL. Subsequently, multi-chain mediation models were implemented to detect whether a chain effect existed between the mediating variables, given the possible interaction between metabolic variables. Mediation hypotheses were tested via a bias-corrected bootstrap method with 5000 samples to calculate 95% confidence intervals (CI). Statistical significance of mediating effects was admitted when zero did not lie within the confidence intervals. Prior to the mediation analysis, we performed multiple linear regression with interaction terms to determine whether there was an interaction between NPs and metabolic variables in the association with LTL. For variables that interacted with NPs, subgroup analyses were performed to further explore the relationship between NPs and LTL across different subgroups.

The statistical analyses were performed by SPSS Windows, version 26.0 (IBM Corp., Chicago, IL, USA) and R software version 4.2. A two-sided P-value < 0.05 was considered statistically significant.

## Results

### Five main nutrient patterns by PCA

We identified five nutrient patterns through PCA analysis, which accounted for 82.36% of the total variance in nutrient intake. These NPs were named based on the factor loadings of nutrients, as indicated in Table S1. The first NP (factor 1) exhibited high loadings of proteins, carbohydrates, fiber, vitamin B1, and several minerals (potassium, magnesium, iron, selenium, zinc, copper), and was termed the “balanced-nutrient” pattern. The second NP (factor 2) was characterized by increased positive loadings of vitamin C, calcium, potassium, magnesium, and fiber. Notably, the loadings for magnesium, fiber, and potassium were elevated in both factors 1 and 2, with the former two being higher in factor 1 and therefore considered only in factor 1, while potassium was higher in factor 2 and thus considered in factor 2. Consequently, factor 2 was named the “high vitamin C-calcium-potassium” pattern. The third NP (factor 3) was dubbed the “high vitamin A-vitamin B2” pattern due to the high consumption of these two nutrients. The fourth NP (factor 4) showed a higher intake of vitamin E and fat and was therefore named the “high vitamin E-fat” pattern. The fifth NP (factor 5), referred to as the “high sodium” pattern, was strongly correlated with sodium intake.

### Baseline characteristics between different LTL groups

Participants in the shorter LTL groups (Q1 and Q2) were significantly older than those in the longer LTL group (Q3 and Q4). Compared to the other three groups, individuals in the shortest LTL group exhibited greater WC, higher blood pressure (both SBP and DBP), FBG, and HbA1c, as well as higher ratios of HTN and DM. Interestingly, regarding blood lipids, levels of TC and HDL-C in the shorter LTL groups (Q1 and Q2) were lower than those in the longer LTL groups (Q3 and Q4), while non-HDL-C showed the opposite trend. Regarding the factor scores of NPs, significant differences also existed in different levels of LTL groups. The shortest LTL group had the lowest scores for factor 1 and the highest scores for factors 3 and 5 compared to the remaining three groups. This suggests that subjects in the former group might exhibit poorer compliance with the “balanced-nutrient” pattern and greater compliance with the “high vitamin A-vitamin B2” and “high sodium” patterns. Factor 2 had the lowest scores in the Q4 group while factor 4 showed the highest scores in that group. There were no significant differences in sex, BMI, sUA, eGFR, TG, LDL-C, HOMA-IR, TG/HDL-C, and the TyG index between different groups. (Table 1)

**Table 1:** The baseline characteristics of participants according to LTL quartiles

	Overall(*n* = 779)	Q1(*n* = 195,<-1.009)	Q2(*n* = 194,1.009 ~ 0.568)	Q3(*n* = 195,0.568 ~ 0.951)	Q4(*n* = 195,>0.951)	*P*
Age(y)	54.92 ± 11.02	57.03 ± 10.79	57.60 ± 10.58)	52.73 (10.85)	52.32 (10.88)	< 0.001*
Female (%)	487 (62.5)	114 (58.5)	121 (62.4)	128 (65.6)	124 (63.6)	0.517
WC(cm)	88.61 (10.61)	91.64 (11.75)	89.30 (10.64)	85.78 (9.42)	87.81 (9.74)	< 0.001*
BMI(kg/m^2^)	26.01 (3.83)	26.34 (4.19)	25.81 (3.61)	25.77 (3.77)	26.14 (3.73)	0.403
SBP(mmHg)	129.69 (18.02)	134.06 (18.60)	130.04 (16.50)	126.41 (17.23)	128.43 (18.85)	< 0.001*
DBP(mmHg)	78.21 (10.98)	81.43 (11.52)	78.74 (11.64)	76.70 (9.68)	76.11 (10.34)	< 0.001*
HTN (%)	304 (39.0)	105 (53.8)	87 (44.8)	54 (27.7)	58 (29.7)	< 0.001*
eGFR(mL/min/1.73m^2^)	95.13 (19.65)	96.99 (15.66)	95.26 (24.23)	93.94 (18.85)	94.28 (18.95)	0.423
UA(umol/L)	293.67 (83.63)	295.36 (86.13)	297.32 (83.49)	299.18 (84.69)	282.73 (79.67)	0.208
TC(mmol/L)	5.12 (1.10)	4.75 (0.94)	4.75 (1.07)	5.40 (1.06)	5.58 (1.05)	< 0.001*
TG(mmol/L)	1.36 (0.96, 2.03)	1.40 (0.97, 1.98)	1.25 (0.84, 2.04)	1.39 (1.02, 2.07)	1.41 (0.98, 2.00)	0.359
HDL-C(mmol/L)	1.27 (0.35)	1.21 (0.30)	1.27 (0.32)	1.31 (0.47)	1.28 (0.26)	0.043*
LDL-C(mmol/L)	2.84 (0.77)	2.84 (0.79)	2.82 (0.85)	2.80 (0.71)	2.89 (0.73)	0.71
Non-HDLC(mmol/L)	3.85 (1.06)	3.53 (0.90)	3.48 (1.00)	4.08 (1.15)	4.30 (0.96)	< 0.001*
FBG(mmol/L)	6.00 (5.40, 7.30)	6.50 (5.40, 8.65)	6.00 (5.30, 7.25)	5.85 (5.42, 6.50)	6.00 (5.51, 6.98)	0.009*
FINS(mU/L)	9.00 (6.05, 12.97)	8.80 (5.58, 12.84)	8.11 (5.48, 11.14)	10.01 (6.72, 14.17)	9.54 (6.71, 13.15)	0.001*
HbA1c(%)	5.70 (5.30, 6.50)	6.20 (5.40, 7.10)	5.70 (5.40, 7.00)	5.70 (5.30, 6.10)	5.60 (5.30, 6.00)	< 0.001*
Glucose state (%)						< 0.001
NGT(%)	298 (38.3)	63 (32.3)	64 (33.0)	92 (47.2)	79 (40.5)	
PreDM(%)	176 (22.6)	18 (9.2)	43 (22.2)	57 (29.2)	58 (29.7)	
DM(%)	305 (39.2)	114 (58.5)	87 (44.8)	46 (23.6)	58 (29.7)	
HOMA-IR	3.34 (3.09)	3.59 (3.59)	3.17 (3.57)	3.31 (2.67)	3.31 (2.33)	0.608
TG/HDL-C	1.10 (0.70, 1.83)	1.14 (0.72, 1.98)	0.99 (0.63, 1.80)	1.13 (0.74, 1.78)	1.13 (0.73, 1.83)	0.506
TyG	8.92 (0.71)	8.99 (0.75)	8.84 (0.74)	8.89 (0.66)	8.95 (0.67)	0.175
Z-LTL	0.00 (1.00)	-1.24 (0.14)	-0.67 (0.34)	0.81 (0.10)	1.09 (0.12)	< 0.001*
Factor1	-0.14 (-0.68, 0.42)	-0.27 (-0.80, 0.36)	-0.24 (-0.74, 0.26)	0.07 (-0.44, 0.72)	-0.14 (-0.64, 0.38)	0.001*
Factor2	-0.22 (-0.47, 0.11)	-0.14 (-0.44, 0.19)	-0.12 (-0.40, 0.27)	-0.26 (-0.47, 0.11)	-0.30 (-0.54, -0.07)	< 0.001*
Factor3	-0.14 (-0.35, 0.16)	0.01 (-0.22, 0.29)	0.00 (-0.23, 0.28)	-0.23 (-0.42, -0.01)	-0.26 (-0.42, -0.04)	< 0.001*
Factor4	-0.32 (-0.68, 0.32)	-0.30 (-0.66, 0.19)	-0.44 (-0.67, -0.13)	-0.22 (-0.70, 0.46)	-0.09 (-0.68, 1.17)	< 0.001*
Factor5	-0.20 (-0.56, 0.41)	0.33 (-0.12, 1.13)	0.30 (-0.18, 0.95)	-0.50 (-0.76, -0.23)	-0.46 (-0.71, -0.22)	< 0.001*
Total Energy(kcal)	1381.00 (1015.88, 1847.05)	1283.93 (961.30, 1771.37)	1168.06 (895.00, 1710.91)	1552.42 (1194.21, 1984.86)	1481.45 (1083.91, 1918.12)	< 0.001*

Abbreviation: BMI: body mass index; WC: waist circumference; SBP: systolic blood pressure; DBP: diastolic blood pressure; HTN: hypertension; sUA: serum uric acid; eGFR: estimated glomerular filtration rate; TC: total cholesterol; TG: triglyceride; HDL-C: high-density lipoprotein cholesterol; LDL-C: low-density lipoprotein cholesterol; non-HDL-C: non-high-density lipoprotein cholesterol; FBG: fasting blood glucose; HbA1c: glycated hemoglobin; FINS: fasting insulin; NGT: normal glucose tolerance; preDM: prediabetes mellitus; DM: diabetes mellitus; HOMA-IR: the homeostatic model assessment of insulin resistance; TG/HDL-C: triglyceride to high-density lipoprotein cholesterol ratio; TyG: triglyceride-glucose; Z-LTL: z scores standardized leukocyte telomere length

Factor 1, the “balanced-nutrient” pattern; Factor 2, the “high vitamin C-calcium-potassium” pattern; Factor 3, “high vitamin A-vitamin B2” pattern; Factor 4, “high vitamin E-fat” pattern; Factor 5, “high sodium” pattern

*P < 0.05 means statistical difference

### The correlation analysis between nutrient pattern scores, LTL, and metabolic parameters

**Fig. 1:**
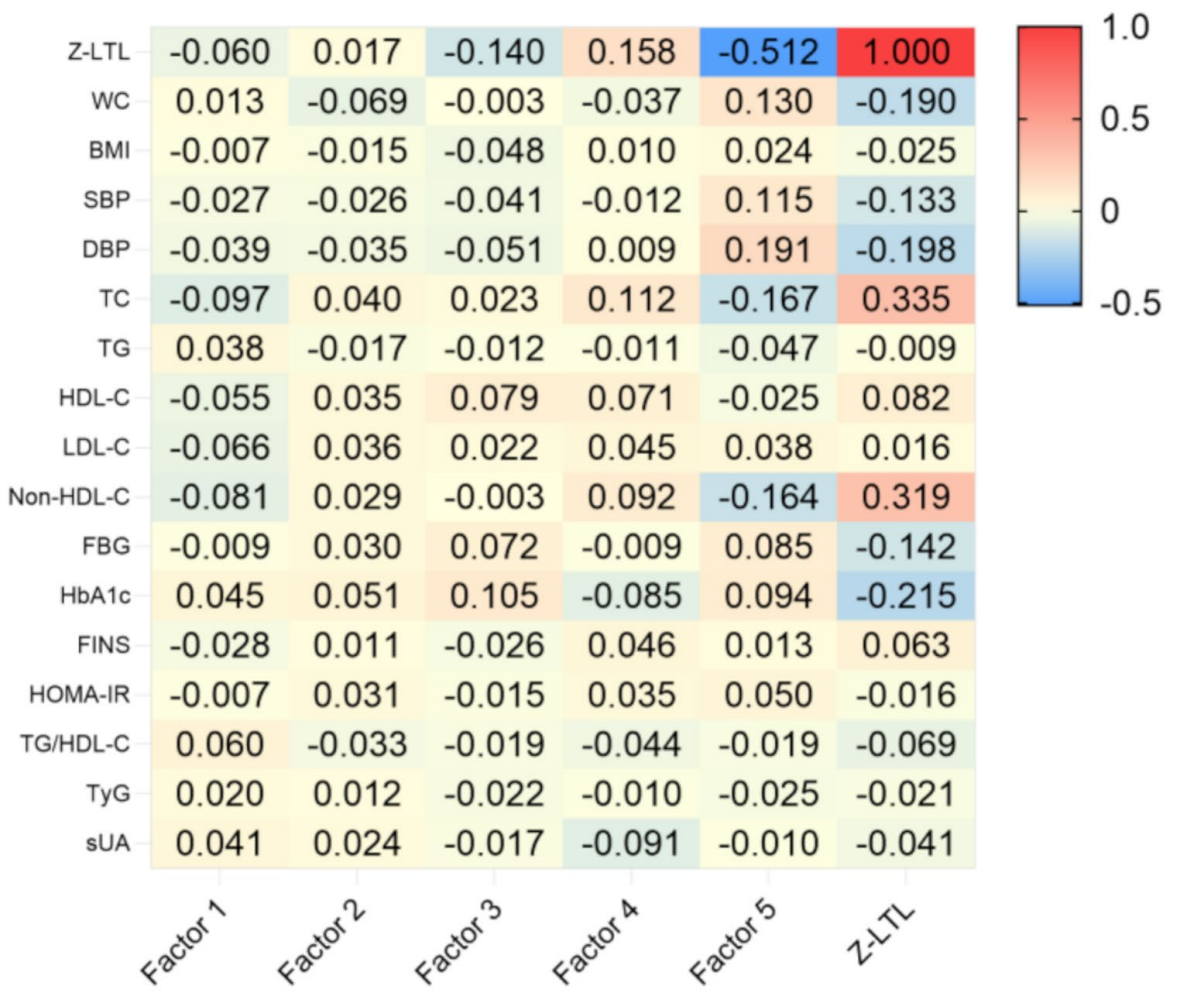
Heat Map of the partial correlation analysis between nutrient pattern scores, z-LTL, and metabolic parameters. The heat map data represent the correlation coefficient’s value, such as an absolute value of *r* > 0.07, *P <* 0.05. The red represents a positive correlation coefficient, the blue represents a negative correlation coefficient, and darker colors represent larger absolute values of the correlation coefficient. Factor 1, the “balanced-nutrient” pattern; Factor 2, the “high vitamin C-calcium-potassium” pattern; Factor 3, “high vitamin A-vitamin B2” pattern; Factor 4, “high vitamin E-fat” pattern; Factor 5, “high sodium” pattern

Spearman correlation analysis showed that all five nutrient patterns (*r* = 0.799, -0.079, -0.368, 0.450, and − 0.377, respectively; all *P* < 0.05) and z-LTL (*r* = 0.119, *P* < 0.05) were significantly associated with total energy intake. Therefore, we proceeded with partial correlation analysis, adjusting for the potential confounding effect of total energy intake, to evaluate the relationships between nutrient patterns, z-LTL, and metabolic variables. The results indicated that, the scores for factor 3 and factor 5 were mildly and moderately negatively correlated with z-LTL, whereas factor 4 exhibited a slight positive correlation after controlling for energy. No correlation with z-LTL was observed for factor 1 and factor 2. Furthermore, the scores for factor 5 were detected to be correlated with multiple metabolic variables, including positive correlation with WC, SBP, DBP, FBG, and HbA1c, and reverse correlation with TC and non-HDL-C. Additionally, among the other NPs related to LTL, factor 4 was positively correlated with TC (including HDL-C and non-HDL-C) and negatively associated with HbA1c and sUA. Factor 3 showed a positive correlation with HDL-C, FBG, and HbA1c. Metabolic parameters negatively associated with z-LTL included WC, SBP, DBP, FBG, and HbA1c, whereas TC and non-HDL-C were positively correlated. (Fig. 1)

### The linear regression of nutrient patterns and LTL in multiple adjusted models

In crude mode (model 1), factor 3 and factor 5 were observed to have a negative association with z-LTL, whereas factor 4 displayed a positive association with z-LTL. After adjusting for the total energy intake, the relationship between the three NPs and z-LTL still persisted (model 2). In model 3, which controlled for age and sex based on model 2, the significant associations between the NPs and z-LTL remained evident, except the correlation with factor 4 was only slightly weakened. Upon further adjustment for multiple potential metabolic confounders in model 4, the link between factor 5 and z-LTL was remarkably attenuated. A similar reduction was noted for factor 4, with factor 3 experiencing only a minor decline. (Table 2) This indicated that metabolic factors might play a role in the relationship between factor 4, factor 5, and LTL. Moreover, no association between factor 1 and factor 2 with z-LTL was still found.

**Table 2:** The multiple linear regression between nutrient patterns and z-LTL

	Factor1	Factor 2	Factor 3	Factor 4	Factor 5
	*B*(95%CI)	*B*(95%CI)	*B*(95%CI)	*B*(95%CI)	*B*(95%CI)
Model 1	0.077(0.007, 0.148)	0.035(-0.036, 0.105)	-0.153(-0.224, -0.082) *	0.201(0.132, 0.270) *	-0.556(-0.619, -0.493) *
Model 2	-0.102(-0.222, -0.017)	0.017(-0.054, 0.087)	-0.141(-0.212, -0.071) *	0.174(0.097, 0.250) *	-0.553(-0.619, -0.488) *
Model 3	-0.109(-0.227, 0.008)	0.009(-0.060, 0.079)	-0.141(-0.210, -0.072) *	0.159(0.084, 0.235) *	-0.534(-0.599, -0.468) *
Model 4	-0.038(-0.148, 0.072)	0.005(-0.059, 0.069)	-0.120(-0.183, -0.057) *	0.099(0.029, 0.170) *	-0.481(-0.549, -0.413) *

Model 1: crude model. Model 2: adjusted for total energy intake. Model 3: model 2 + age and sex. Model 4: adjusted for WC, SBP, DBP, TG, HDL-C, non-HDL-C, HbA1c, sUA, and FINS based on model 3

Abbreviation: WC: waist circumference; SBP: systolic blood pressure; DBP: diastolic blood pressure; TC: total cholesterol; TG: triglyceride; HDL-C: high-density lipoprotein; LDL-C: low-density lipoprotein; non-HDL-C: non-high-density lipoprotein; FBG: fasting blood glucose; HbA1c: glycated hemoglobin; sUA: serum uric acid; FINS: fasting insulin; Z-LTL: z scores standardized leukocyte telomere length

Factor 1, the “balanced-nutrient” pattern; Factor 2, the “high vitamin C-calcium-potassium” pattern; Factor 3, “high vitamin A-vitamin B2” pattern; Factor 4, “high vitamin E-fat” pattern; Factor 5, “high sodium” pattern

*P < 0.05 means statistical difference

### Mediated effects of metabolism factors on the correlation between nutrient patterns and LTL

Mediation models were employed to investigate whether metabolic factors can mediate the effect of factor 4 and factor 5 on LTL. Guided by clinical practice and the correlation analysis findings, we screened for potential metabolic variables, including WC, SBP, DBP, HbA1c, TG, non-HDL-C, and HDL-C, then conducted individual simple mediation models, respectively. Before conducting the mediation analysis, multiple linear regression with interaction terms was performed to identify the interaction between factors 4 and 5 and metabolic variables in the association with LTL. The results indicated that only factor 5 and non-HDL-C had significant interaction (*P* < 0.001), prompting the exclusion of non-HDL-C from further mediation analysis. (Table S2 and Fig. S2)

Mediation analysis revealed that WC and DBP could mediate the association between factor 5 and LTL, with an indirect effect not involving zero. For factor 4, the mediators identified were HbA1c and non-HDL-C, highlighting their role in the relationship with LTL. However, other metabolic indicators were not observed to play mediating roles in the relationship between factor 4, factor 5, and LTL (Table S3).

**Fig. 2:**
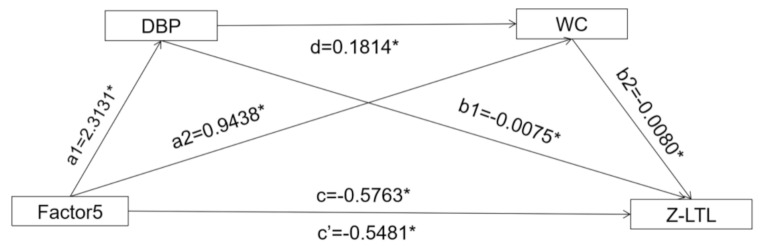
Association of the factor 5 and z-LTL mediated by DBP and WC. a1 represents the effect of factor5 on DBP (*B* = 2.3131(95%CI(1.3996,3.2266), *P* < 0.0001); a2 represents the effect of factor5 on WC (*B* = 0.9438 (95%CI (0.0853, 1.8024), *P* = 0.0312); b1 represents the effect of DBP on z-LTL (*B*=-0.0075 (95%CI (-0.0132,-0.0018), *P* = 0.0097); b2 represents the effect of WC on z-LTL (*B*=-0.0080(95%CI (-0.0140,-0.0019), *P* = 0.0096); d represents the effect of DBP on WC (*B* = 0.1814(95%CI (0.1144,0.2483), *P* < 0.0001); c represents the total effect (*B*=-0.5763(95%CI(-0.6472,-0.5054), *P* < 0.0001); c’ represents the direct effect (*B*=-0.5481(95%CI (-0.6197,-0.4765), *P* < 0.0001). Model was adjusted for energy, age, and gender. DBP: diastolic blood pressure; WC: waist circumference; Z-LTL: z scores standardized leukocyte telomere length; Factor 5: “high sodium” pattern. * *P* < 0.05 means statistical difference

Subsequently, we further conducted a chain-mediating model to investigate the co-mediating role of DBP and WC could play in the relationship of factor 5 and LTL, as depicted in Fig. 2; Table 3. The findings revealed that DBP and WC not only independently mediated the association between “high sodium” pattern and LTL (*B*_DBP_=-0.0173(-0.0333, -0.0041), *B*_WC_= -0.0075(-0.0186, -0.0004), all *P* < 0.05), with the mediation proportion 3.00% and 1.30%, respectively; the co-mediation of them was also statistically significant (*B*_joint_=-0.0033(-0.0072, -0.0006)).

**Table 3:** The mediating effect and proportion of DBP and WC on the association between factor 5 and z-LTL

Model pathway	*B*(95%CI)	Proportion mediated(%)
Total effect	-0.5763(-0.6472, -0.5054) *	-
Indirect effect by DBP	-0.0173(-0.0333, -0.0041) *	3.00%
Indirect effect by WC	-0.0075(-0.0186, -0.0004) *	1.30%
Indirect effect by DBP and WC	-0.0033(-0.0072, -0.0006) *	0.57%

The model was adjusted for total energy intake, age, and sex. Factor 5, “high sodium” pattern. Z-LTL, z scores standardized leukocyte telomere length. * *P* < 0.05means statistical difference

Given the observed interaction between non-HDL-C and factor 5 in their association with LTL, we performed a subgroup analysis stratified by tertiles of non-HDL-C levels. The results revealed that increases in non-HDL-C levels intensified the negative relationship between the “high sodium” dietary pattern and LTL within a certain threshold (< 4.22 mmol/L). Beyond this threshold (> 4.22 mmol/L), the negative association began to diminish, albeit it remained more pronounced than at the lowest non-HDL-C levels (< 3.40 mmol/L). (Table S4) These findings indicated that non-HDL-C plays a complex role in modulating the impact of the “high sodium” dietary pattern on LTL.

Besides, we also investigated the separate and combined-mediating effect of HbA1c and non-HDL-C on the relationship between factor 4 and LTL. HbA1c and non-HDL-C can be independent mediators on the positive association between factor 4 and LTL respectively (*B*_HbA1c_=0.0170(0.0010, 0.0347), *B*_non−HDL−C_=0.0335(0.0067,0.0626), all *P* < 0.05); however, co-mediating effect of them did not achieve statistical significance (*B*_joint_=-0.0024(-0.0061,0.0001)). (Fig. S3 and Table S5)

## Discussion

The study evaluated the association of nutrient-based dietary patterns with LTL and explored the potential mediating roles of metabolic factors among them within the Chinese population. It found that a “high sodium” nutrient pattern was inversely correlated with LTL, where DBP and WC could serve as individual and combined mediators in this relationship. Besides, the NP rich in vitamin E and fat showed a positive association with LTL, whereas those high in vitamins A and B2 were negatively associated with LTL.

High sodium consumption has been definitively linked to an increased risk of age-related diseases, including hypertension, cardiovascular disease, and stroke [24, 25]. Research by Zhu et al. demonstrated a correlation between elevated sodium intake and reduced LTL in overweight and obese adolescents [8]. Myers’ study also showed an inverse association between maternal sodium intake and fetal TL [26]. Our findings further support these observations, indicating a detrimental association between a high-sodium dietary pattern and LTL. Such uniformity in results underscores the potential harm of excessive sodium intake on the maintenance of TL. Furthermore, linear regression model revealed that the relationship between sodium intake and LTL weakened after adjusting for metabolic factors. Given that metabolic indicators, such as BP, WC, HbA1c, and non-HDL-C, were linked to both high sodium consumption and LTL, we hypothesized that metabolic factors might influence this association. Subsequent mediation analyses indicated that DBP and WC independently and collectively mediates the impact of a high-sodium diet on LTL, suggesting adverse effects of high sodium intake on LTL through the augmentation of DBP, WC, and their combined effects. Echoing these findings, Rehkopf identified significant inverse relationships between DBP, WC, and LTL by examining 17 cardiovascular risk factors [27]. A decade-long longitudinal study also found that an increase in WC was associated with faster telomere shortening [28]. Additionally, both empirical and clinical evidence suggested that hypertension could lead to telomere shortening, while reducing BP may protect against telomere length shorten [29, 30]. The most recent research highlights that the DASH diet, known for its sodium restriction, could be beneficial for hypertension by lowering the waist-to-height ratio [31]. These consistent findings supported the role of metabolic dysfunctions, such as elevated BP and central obesity, in accelerating telomere attrition due to a high sodium diet. The effect of high sodium on telomeres may not only be through a single metabolic factor but also the interaction of metabolic factors.

In addition, it should be noted that we also found a nuanced interaction of non-HDL-C on the association between high sodium diet and telomere. Within a certain threshold, elevated levels of non-HDL-C could intensify the negative effects of a high sodium diet on LTL. However, beyond a specific point (non-HDL-C levels ≥ 4.22mmol/L), this association begins to diminish. This suggests that the influence of non-HDL-C on the relationship between a high sodium intake and telomere length may be dose-dependent. The intricate contribution of lipid metabolism to the dynamics between a high sodium diet and LTL remains to be fully understood, calling for further comprehensive research to elucidate these complexities.

Besides, we identified a positive linear correlation between a high “vitamin E-fat” nutrient pattern and LTL, suggesting a potential protective role of this NP on LTL, possibly through the reduction of HbA1c levels and an increase in non-HDL-C as indicated by mediation analysis. Prior research has highlighted the benefits of vitamin E in modulating LTL, attributing these effects to its antioxidant and anti-inflammatory properties [32, 33]. Additionally, it has been demonstrated that increasing dietary intake of vitamin E can significantly lower HbA1c levels and raise non-HDL-C concentrations [34, 35], which supported our findings that dietary vitamin E may influence telomeres by inducing metabolic alterations. The deleterious effect of blood glucose on telomeres has been well-documented [36], yet the exploration of blood lipids and LTL has been limited and yields conflicting results [28, 37]. Our study primarily revealed a correlation between blood lipids and LTL through non-HDL-C, diverging from previous research that focused on LDL-C and HDL-C. This suggests the potential involvement of other cholesterols in LTL maintenance. Currently, the mechanism of action of cholesterol on telomeres has not been fully elucidated, with the differential impacts of various cholesterols on telomerase posited as a possible explanation [38, 39]. It is also noteworthy that fat contributed significantly to this nutrient pattern. Some studies suggested that total fat intake was unrelated to LTL; by contrast, fatty acids were more prone to be associated with LTL, depending on their type and proportion [7, 40, 41]. Dietary fats can be divided into a variety of fatty acids, whose effects on telomeres are complicated. In the future, it’s necessary to refine the classification of dietary fats and fatty acids to explore their effects on telomeres and underlying mechanisms.

In the study, NP rich in vitamin A and vitamin B2 was found to have a slight negative correlation with LTL, whereas no significant relationship was identified between high “vitamin C-calcium-potassium” NP and LTL. The scientific community has extensively investigated the links between individual micronutrients and LTL, yielding mixed outcomes. For instance, some studies found that fiber, vitamin C, vitamin A, copper, and potassium were positively correlated with LTL, while others reported that vitamin A, vitamin B2, and calcium were not associated with that [6, 42]. Besides, our study also failed to detect the relation between the “balanced-nutrient” pattern–characterized by carbohydrates, protein, fiber, and multiple micro-nutrients like zinc, magnesium, iron, copper, and LTL. These nutrients have been shown a different correlation with LTL in earlier findings [7, 19, 43, 44]. As mentioned above, various nutrients possess diverse effects on LTL, and we suspected that intricate interactions between nutrients may alter the effect of individual nutrients, which may explain the difference between our study and others. However, our study sample was relatively small, and future larger studies are required to explore the association between nutrient combinations and telomeres.

Multiple pieces of evidence support that environmental variables such as diet and exercise are important factors influencing LTL. However, recent research from the UK Biobank suggests that the association between modifiable behaviors such as diet and LTL is modest. This minor impact may not be sufficient to significantly alter the relationship between LTL and various diseases or life expectancy [45, 46]. Our study also found that among the three dietary patterns associated with LTL, these correlation coefficients were only mild to moderate, similar to the findings of the aforementioned study. This indicates that changing dietary patterns may be beneficial for slowing telomere attrition, but the clinical benefits are relatively small, and whether it can alter adverse outcomes of major diseases related to telomere shortening remains to be further explored.

Besides, substantial evidence has demonstrated that an increase in inflammation and oxidative stress, which are influenced by diet and metabolic factors, plays a crucial role in telomere shortening [47]. Our research indicates that the indirect effect of metabolic disorders on the relationship between nutrient patterns and LTL was also minor, leading us to hypothesize the presence of other mediating factors, such as inflammation and oxidative stress, beyond metabolism. Regrettably, this study lacked indicators for these potential mediators. Therefore, the complex interplay among dietary nutrient patterns, metabolism, inflammation, oxidative stress, and telomeres warrants further investigation in future research.

### Strengths and limitations

This study has several advantages. It represents the inaugural investigation into the association between dietary patterns of nutrients and LTL in a Chinese population, unveiling the intricate impact of nutrient interrelations on LTL. Furthermore, this is the first study to dissect the varied influence of metabolic factors on the nexus between nutrient patterns and LTL, employing mediation models for this exploration. Additionally, the focus on nutrient-based dietary patterns, as opposed to food groups, facilitates more straightforward guidance from nutritionists to the public, with the universality of nutrients allowing for comparisons across different ethnic groups.

However, there were also several limitations. Firstly, it was a cross-sectional design from which we cannot infer causality, thus, our findings need to be further confirmed from prospective studies. Secondly, the 24-hour food recalls may not accurately capture long-term nutritional intake, especially considering the unique and complex dietary habits of the Chinese population, which may not align well with the methodologies used in food frequency questionnaires (FFQ). Additionally, while the PCA method employed to identify nutrient patterns effectively reflects real-world dietary behaviors, it relies on subjective choices in naming and selecting nutrients, potentially introducing measurement errors. Notably, compared to sodium intake measured by dietary questionnaires, the 24-hour urinary sodium level may offer a more accurate reflection of actual sodium consumption. Future research should integrate this indicator for a more comprehensive assessment of sodium intake. Thirdly, the sample size was relatively small and from a single geographic location, which may somewhat limit the generalizability of the results. Additionally, variables that could affect LTL, such as social deprivation, race, and white blood cell count, were not included in our analysis, which could also influence the final results.

## Conclusions

This study indicated that the “high sodium” pattern was adversely linked with LTL, with SBP and WC acting as potential mediators, either individually or in combination. Besides, a “high vitamin E-fat” pattern showed a beneficial association with LTL, possibly through lowering HbA1c and increasing non-HDL-C. Different nutrient patterns showed complex associations with metabolism and aging. Future large-scale prospective studies are needed to elucidate these associations and underlying mechanisms, to recommend more appropriate dietary suggestions for the public.

## Electronic supplementary material

Below is the link to the electronic supplementary material.

Supplementary Material 1: Fig. S1 Flow chart of study participants included. Table S1 Orthogonally rotated factor loadings for the five nutrient patterns^a^. Table S2 Interaction effect between high vitamin E-fat, high sodium pattern, and metabolic indicators in the association with z-LTL. Fig. S2. Interaction between factor 5 and non-HDL-C in the association with LTL. Table S3 Mediation effect of metabolic indicators on the relationship between high vitamin E-fat and high sodium pattern and z-LTL. Table S4 Multiple linear regression between factor 5 and z-LTL in non-HDL-C tertiles groups. Fig. S4 Association of the high fat pattern and z-LTL mediated by HbA1c and non-HDL-C. Table S5 The mediating proportion of HbA1c and non-HDL-C on the association between factor 4 and z-LTL

## Data Availability

No datasets were generated or analysed during the current study.
