# Supplementary material for: The negative association between sodium-driven nutrient pattern and telomere length: the chain mediating role of diastolic pressure and waist circumference
Source: Aging Clin Exp Res. 2024 Oct 5;36(1):201. doi: 10.1007/s40520-024-02852-1 (PMC11455806; doi:10.1007/s40520-024-02852-1)
Supplement: Supplementary file 1 — Supplementary Material 1: Fig. S1 Flow chart of study participants included. Table S1 Orthogonally rotated factor loadings for the five nutrient patternsa. Table S2 Interaction effect between high vitamin E-fat, high sodium pattern, and metabolic indicators in the association with z-LTL. Fig. S2. Interaction between factor 5 and non-HDL-C in the association with LTL. Table S3 Mediation effect of metabolic indicators on the relationship between high vitamin E-fat and high sodium pattern and z-LTL. Table S4 Multiple linear regression between factor 5 and z-LTL in non-HDL-C tertiles groups. Fig. S4 Association of the high fat pattern and z-LTL mediated by HbA1c and non-HDL-C. Table S5 The mediating proportion of HbA1c and non-HDL-C on the association between factor 4 and z-LTL [file 40520_2024_2852_MOESM1_ESM.docx]

The negative association between sodium-driven nutrient pattern and telomere length: the chain mediating role of diastolic pressure and waist circumference(**Supplementary materials**)

Baodi Xing^1^, Jie Yu^1^, Yiwen Liu^1^, Shuli He^2^, Qi Gao^1^, Xinyue Chen^1^, Fan Ping^1^, Lingling Xu^1^, Wei Li^1^, Hubing Zhang^1*^, and Yuxiu Li^1*^

Page2: Fig. S1

Page3: TableS1

Page4: Table S2 and Fig.S2

Page5: Table S3 and Table S4

Page6: Fig. S2 and Table S5

**
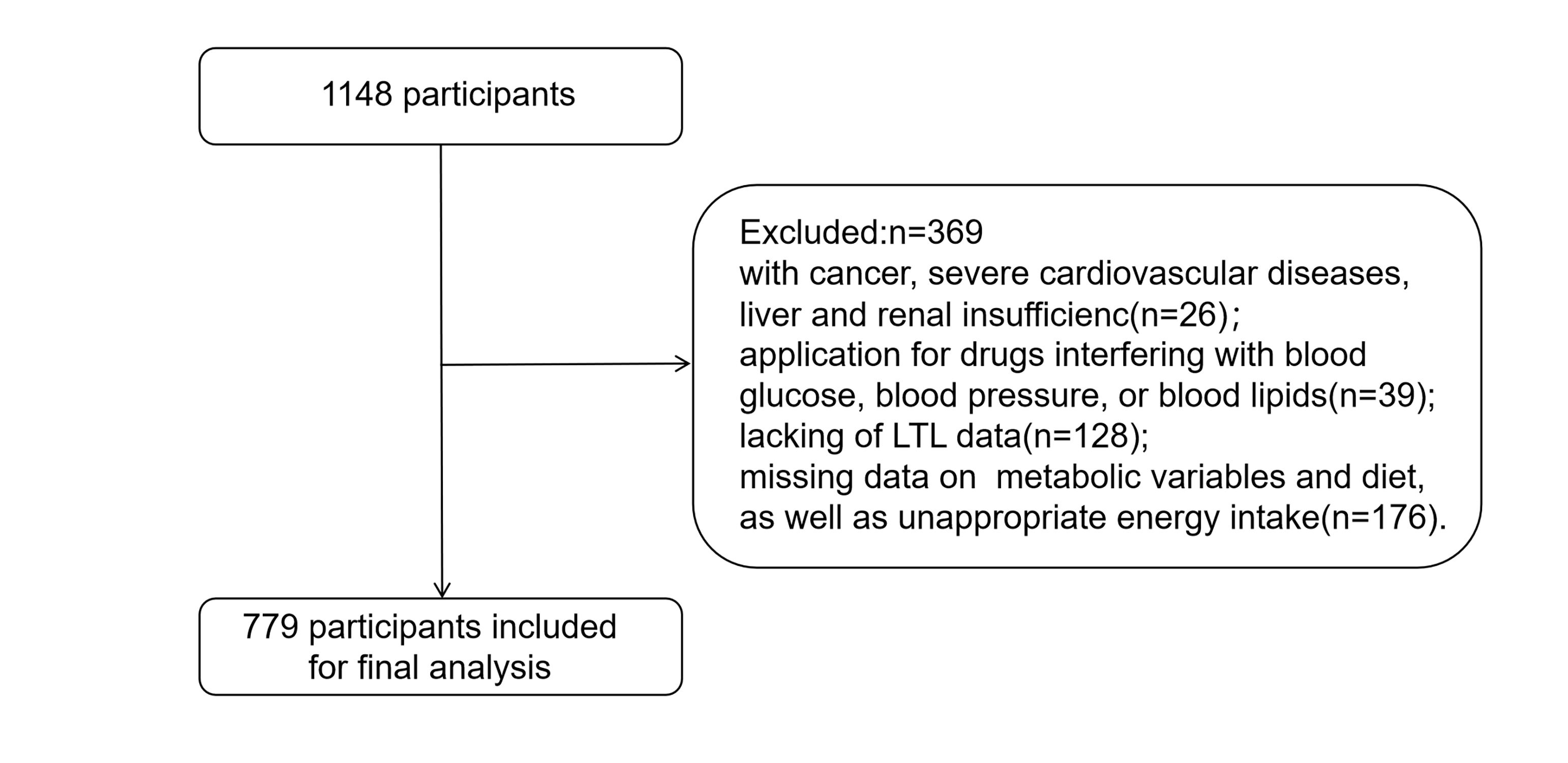
**

**Fig. S1** Flow chart of study participants included.

**Table S1** Orthogonally rotated factor loadings for the five nutrient patterns^a^

|  | Factor1 | Factor2 | Factor3 | Factor4 | Factor5 |
| --- | --- | --- | --- | --- | --- |
| Protein | **0.866** | 0.082 | 0.233 | 0.161 | 0.251 |
| Carbohydrate | **0.849** | 0.177 | -0.074 | 0.035 | -0.108 |
| Fat | 0.250 | 0.045 | 0.017 | **0.847** | 0.251 |
| Fiber | **0.667** | **0.543** | 0.081 | 0.014 | 0.026 |
| VitaminA | 0.040 | 0.049 | **0.928** | 0.034 | -0.064 |
| VitaminB1 | **0.832** | 0.196 | 0.043 | 0.130 | -0.066 |
| VitaminB2 | 0.392 | 0.117 | **0.738** | 0.200 | 0.196 |
| VitaminC | 0.129 | **0.905** | 0.016 | 0.035 | 0.056 |
| VitaminE | 0.049 | 0.012 | 0.148 | **0.901** | -0.137 |
| Calcium | 0.313 | **0.864** | 0.081 | -0.002 | 0.107 |
| Potassium | **0.559** | **0.772** | 0.085 | 0.046 | 0.059 |
| Magnesium | **0.760** | **0.557** | 0.071 | 0.041 | 0.042 |
| Sodium | 0.020 | 0.128 | 0.024 | 0.044 | **0.921** |
| Iron | **0.680** | 0.441 | 0.282 | 0.091 | 0.068 |
| Selenium | **0.711** | 0.035 | 0.238 | 0.047 | 0.366 |
| Zinc | **0.824** | 0.369 | 0.189 | 0.192 | 0.100 |
| Copper | **0.755** | 0.347 | 0.124 | 0.132 | -0.086 |
| Eigenvalue | 8.545 | 1.879 | 1.290 | 1.210 | 1.074 |
| % of Variance | 50.27% | 11.06% | 7.59% | 7.12% | 6.32% |
| Cumulative% | 50.27% | 61.33% | 68.92% | 76.04% | 82.36% |

^a^Factor loadings ≥ 0.47 were considered to have a strong relation with the nutrient pattern.

Factor 1, the “balanced-nutrient” pattern; Factor 2, the “high vitamin C-calcium-potassium” pattern; Factor 3, “high vitamin A-vitamin B2” pattern; Factor 4, “high vitamin E-fat” pattern; Factor 5, “high sodium” pattern.

**Table S2** Interaction effect between high vitamin E-fat, high sodium pattern, and metabolic indicators in the association with z-LTL

|  | Factor 4 | | Factor 5 | |
| --- | --- | --- | --- | --- |
| Interaction effect | *B* | *P* for interaction | *B* | *P* for interaction |
| WC | 0.004 | 0.182 | 0.002 | 0.515 |
| SBP | -0.0004 | 0.828 | -0.004 | 0.054 |
| DBP | 0.001 | 0.782 | -0.005 | 0.117 |
| HbA1c | -0.045 | 0.094 | 0.0001 | 0.995 |
| Non-HDL-C | -0.047 | 0.122 | -0.119 | <0.001* |
| TG | 0.028 | 0.092 | 0.033 | 0.106 |
| HDL-C | -0.147 | 0.143 | -0.175 | 0.097 |

*B* represents regression coefficient of interaction effect .

Abbreviation: WC, waist circumference; SBP, systolic blood pressure; DBP, diastolic blood pressure; HbA1c, glycated

hemoglobin; non-HDL-C, non-high-density lipoprotein; TG, triglyceride; HDL-C, high-density lipoprotein; Z-LTL,

z scores standardized leukocyte telomere length.

Factor 4, “high vitamin E-fat” pattern; Factor 5, “high sodium” pattern.

* *P*<0.05 means statistical difference.


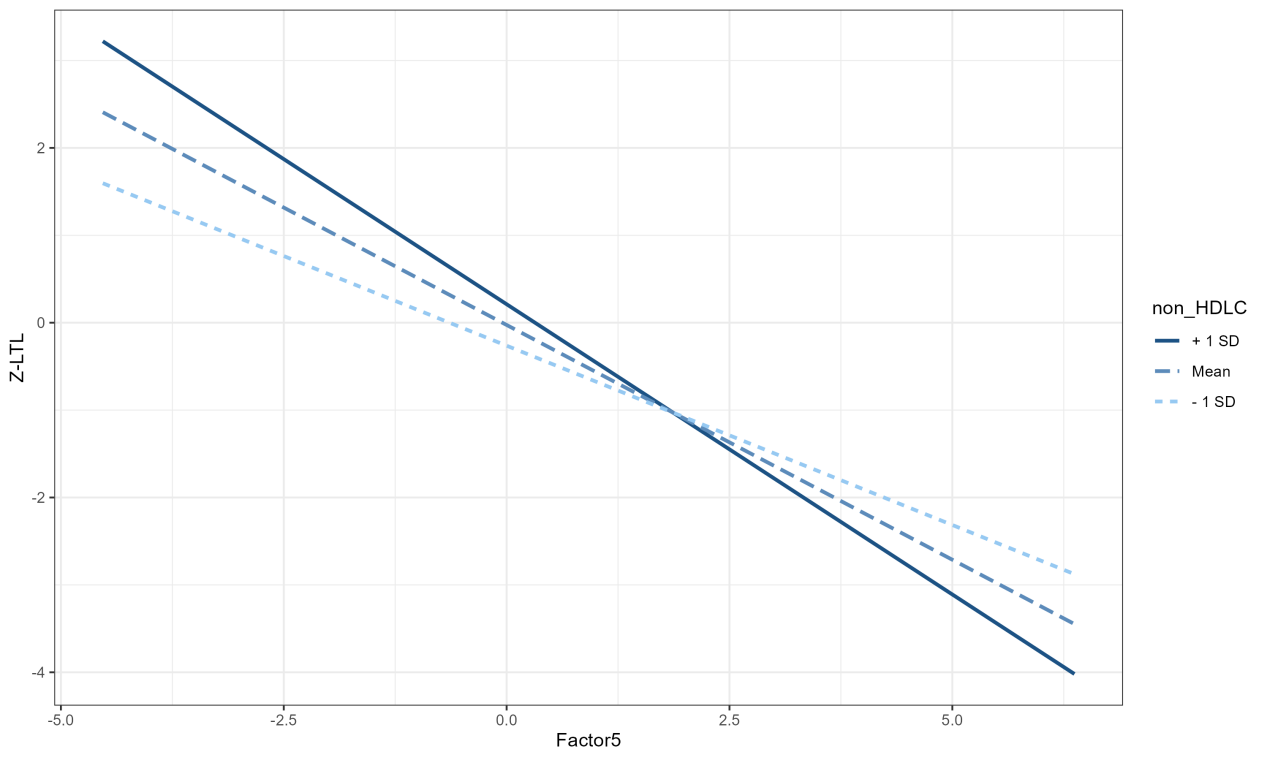


**Fig. S2**. Interaction between factor 5 and non-HDL-C in the association with LTL. | Z-LTL,z scores standardized leukocyte telomere length. Factor 5, “high vitamin E-fat” pattern.

**Table S3** Mediation effect of metabolic indicators on the relationship between high vitamin E-fat and high sodium pattern and z-LTL.

|  | Factor 4 | Factor 5 |
| --- | --- | --- |
| Mediation effect | *B*(95%CI) | *B*(95%CI) |
| WC | 0.002(-0.010,0.014) | -0.011(-0.024,-0.002)* |
| SBP | -0.001(0.076,0.228) | -0.003(-0.012,0.002) |
| DBP | -0.004(-0.020,0.012) | -0.021(-0.037,-0.007)* |
| HbA1c | 0.014(0.001,0.031)* | -0.011(-0.025,0.002) |
| Non-HDL-C | 0.030(0.005,0.057)* | - |
| TG | 0.000(-0.002,0.007) | 0.002(-0.002,0.009) |
| HDL-C | 0.004(0.000,0.012) | -0.001(-0.007,0.005) |

Abbreviation: WC, waist circumference; SBP, systolic blood pressure; DBP, diastolic blood pressure; HbA1c, glycated

hemoglobin; non-HDL-C, non-high-density lipoprotein; TG, triglyceride; HDL-C, high-density lipoprotein; Z-LTL,

z scores standardized leukocyte telomere length.

Factor 4, “high vitamin E-fat” pattern; Factor 5, “high sodium” pattern.

* *P*<0.05 means statistical difference.

**Table S4** Multiple linear regression between factor 5 and z-LTL in non-HDL-C tertiles groups.

| Non-HDL-C(mmol/L) | *B*(95%CI) | *P* |
| --- | --- | --- |
| <3.40 | -0.345 (-0.454, -0.235) | <0.001* |
| 3.40-4.22 | -0.626 (-0.753, -0.500) | <0.001* |
| ≥4.22 | -0.522 (-0.673, -0.431) | <0.001* |

The model was adjusted for WC, SBP, DBP, TG, HDL-C, non-HDL-C, HbA1c, sUA, and FINS based on model 3.

Non-HDL-C, non-high-density lipoprotein cholesterol; Z-LTL, z scores standardized leukocyte telomere length; factor 5, “high sodium” pattern.

* *P*<0.05 means statistical difference.


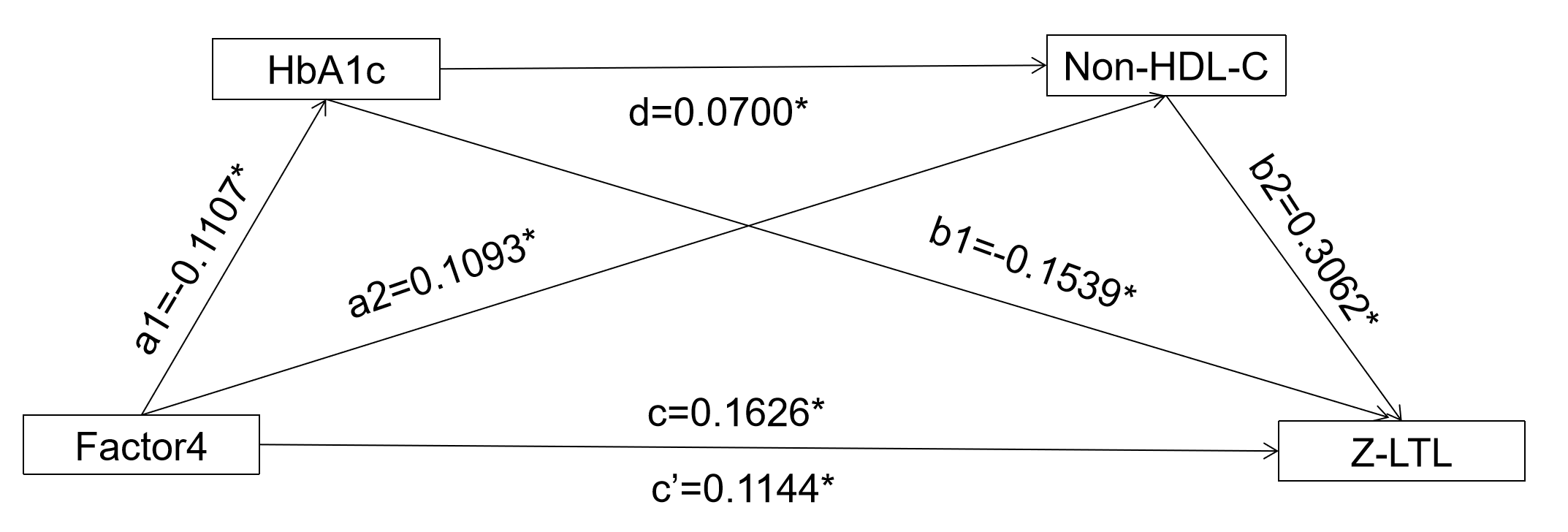


**Fig. S3** Association of factor 4 and z-LTL mediated by HbA1c and non-HDL-C.

a1 represents the effect of factor4 on HbA1c(*B*=-0.1107(95%CI(-0.2111,-0.0103), *P*=-0.0308); a2 represents the effect of factor4 on non-HDL-C(*B*=0.1093(95%CI(0.0256,0.1930), *P*=0.0106); b1 represents the effect of HbA1c on z-LTL (*B*=-0.1539 (95%CI(-0.2044, -0.1034), *P*<0.001); b2 represents the effect of non-HDL-C on z-LTL (*B*=-0.3062(95%CI(-0.2457,-0.3668), *P*<0.001); d represents the effect of HbA1c on non-HDL-C (*B*=0.070(95%CI(0.0107,0.1293), *P*=0.0207); c represents the total effect (*B*=0.1626(95%CI(0.0862, 0.2390), *P*<0.001); c’ represents the direct effect(*B*=0.1144(95%CI(0.0430,0.1858), *P*=0.0017). Model was adjusted for energy, age,and gender.

HbA1c, glycated hemoglobin; non-HDL-C, non-high-density lipoprotein cholesterol;Z-LTL, z scores standardized leukocyte telomere length; factor4: high vitamin E-fat pattern.

* *P*<0.05 means statistical difference.

**Table S5** The mediating proportion of HbA1c and non-HDL-C on the association between factor 4 and z-LTL.

| Model pathway | *B*(95%CI) | Proportion mediated(%) |
| --- | --- | --- |
| Total effect | 0.1626(0.0862,0.2390)* | - |
| Indirect effect by HbA1c | 0.0170(0.0010,0.0347)* | 10.46% |
| Indirect effect by non-HDL-C | 0.0335(0.0067,0.0626)* | 20.60% |
| Indirect effect by HbA1c and non-HDL-C | -0.0024(-0.0061,0.0001) | - |

The model was adjusted for total energy intake, age, and gender.

HbA1c, glycated hemoglobin; non-HDL-C, non-high-density lipoprotein cholesterol;Z-LTL, z scores standardized leukocyte telomere length; Factor4: high vitamin E-fat pattern.

* *P*<0.05 means statistical difference.
